# Participants’ and physiotherapists’ views on the delivery of a randomised controlled feasibility trial investigating a bespoke dynamic elastomeric fabric orthosis for women with chronic pelvic girdle pain: a qualitative exploration

**DOI:** 10.1186/s40814-026-01847-1

**Published:** 2026-06-05

**Authors:** Bradley Halliday, Jennifer Freeman, Sarah Chatfield, Lee Cameron, Kirsty Carter, Jo Hosking, Jill Shawe

**Affiliations:** 1 Faculty of Health, University of Plymouth, Intercity Place, North Road East, Plymouth, PL4 6AB England; 2 Aneurin Bevan University Health Board, Gwent, Wales; 3 Cornwall Partnership NHS Foundation Trust, Cornwall, England; 4 Royal Cornwall Hospital NHS Trust, Cornwall, England

**Keywords:** Pelvic girdle pain, Rehabilitation, Feasibility, Qualitative

## Abstract

**Background:**

Chronic pregnancy-related pelvic girdle pain (PPGP) affects approximately 10–30% of postpartum women beyond 3 months. Such pain is typically recalcitrant to standard care. A dynamic elastomeric fabric orthosis (DEFO) is one option. A feasibility randomised controlled trial (fRCT) assessed the feasibility and acceptability of trial design, interventions and data collection. The aim of this qualitative sub-study was to explore the experience of women participating in the feasibility trial, and physiotherapists delivering the trial intervention.

**Methods:**

An embedded qualitative study within a fRCT. The fRCT randomised participants to either control (standard care involving advice and exercise) or intervention (standard care and DEFO), across three healthcare trusts in three regions of England. Delivery of all interventions and data collection was undertaken remotely. Participants were encouraged to wear the DEFO for a maximum of 12 h a day over the 24-weeks of the trial. The qualitative approach involved online, semi-structured interviews supported by a topic guide. Data was recorded, transcribed verbatim, and analysed using thematic analysis.

**Results:**

Seven (*n* = 5 intervention, *n* = 2 control) of 24 participants recruited to the fRCT and five of the seven physiotherapists involved in the trial intervention delivery participated. Four main themes were identified: “acceptability of trial methods”, “intervention acceptability”, “impact of intervention” and “adherence to exercise”. Data collection methods were acceptable using the web-based app. The virtual nature of the trial was acceptable; however, women and physiotherapists would have preferred at least one face-to-face intervention session. The DEFO was acceptable to women, providing them with a sense of support “holding them together”. They felt it increased awareness of their ability to move, enabling them to be more physically active. Physiotherapists’ felt the process of delivery of the DEFO was acceptable but felt the range of available exercises they could prescribe was restrictive. Physiotherapists and participants found the advice component required further development. Participants struggled to maintain adherence to the prescribed exercise programme over the 24-week period.

**Conclusions:**

The remote trial procedures and interventions were generally acceptable to both participants and physiotherapists, supporting the overall trial design and implementation, but a future design should incorporate an in-person treatment component. The participant’s experiences and suggestions will be considered in the design and delivery of a future definitive trial.

**Trial registration:**

ISRCTN67232113.

**Supplementary Information:**

The online version contains supplementary material available at 10.1186/s40814-026-01847-1.

## Key messages regarding feasibility

- What uncertainties existed regarding the feasibility?
  - Physiotherapists highlighted the key role of primary care in recruiting participants to a future trial, However, there is uncertainty about how this can be best achieved.
- What are the key feasibility findings?
  - The DEFO was acceptable to wear but adherence to wear time varied and was often limited to targeted activities.
  - The remote trial procedures were generally acceptable to participants and physiotherapists, although the advice component requires improvement.
  - Physiotherapists and participants would prefer at least one face-to-face intervention session (preferably the first session).
- What are the implications of the feasibility findings for the design of the main study?
  - Further research is needed to determine optimal recruitment strategies for women with pregnancy-related pelvic girdle pain to a future trial.
  - DEFO wear time guidance needs further consideration to define what is acceptable.
  - Further refinement of data collection processes is warranted, including the timing and frequency of participant reminders.
  - A hybrid or preference-based design may be preferable, which may enhance satisfaction with, and adherence to, the intervention.

## Background

Pregnancy-related pelvic girdle pain (PPGP) is a painful musculoskeletal condition affecting up to 70% of antepartum women [1], with 30% continuing to experience it beyond three months postpartum [2]. A proportion will experience pain for more than ten years postpartum [3]. PPGP has significant socioeconomic; physical [4, 5]; and psychological consequences, with one in four women in the peripartum period experiencing a mental health condition [6].

Current National Institute for Health and Care Excellence (NICE) guidance for the management of PPGP only extends up to eight weeks postpartum [7]. Treatment options for the condition such as exercise and manual therapy have demonstrated limited success [8, 9]. Therefore, there is an urgent need to inform evidenced-based decision-making for women with PPGP.

Pelvic orthotics are an adjunct to treatment, recommended by the European Guidelines [10] and the Royal College of Obstetricians and Gynaecologists [11]. Off-the-shelf orthotics are designed to reduce pain and improve function by providing pelvic support. However, they can compromise movement and be uncomfortable [12]. To overcome this, a bespoke (custom measured) dynamic elastomeric fabric orthosis (DEFO) was designed [13], aiming to provide multiaxial support and sensory input; increasing stability, alignment [14] and potentially maximising movement availability in comparison to off-the-shelf orthotics.

One study has demonstrated that DEFO’s appear more effective than typical “off the shelf” belts in the antepartum period [15] and have shown a stabilising of symptoms in postpartum women [16]. However, the acceptability and impact of pelvic DEFO’s for postpartum women with chronic PPGP is unknown and requires further study [17, 18]. To begin to address this, we undertook a feasibility randomised controlled trial (fRCT) comparing standard care (standardised advice and exercise) with standard care and a DEFO, with an embedded qualitative study. The intention was to determine the feasibility and acceptability of key aspects of the trial (from participants and physiotherapists).

This embedded qualitative study within the fRCT [19] explored:

- Acceptability of trial methods
- Acceptability (comfort, wear-time) of the DEFO
- The impact the intervention may/may not have on women’s lives
- Adherence to the exercise regime over the course of the trial

## Methods

This fRCT, delivered remotely, involved 24 women with severe, chronic PPGP (causing walking or stair climbing to be bothersome and lasting more than three months). It was conducted over three sites in geographically different regions of England. The study protocol [19] and results have been published [20]. Here we focus on reporting the embedded qualitative study described in line with qualitative research reporting standards [21].

### Patient and public involvement (PPI)

Women with PPGP were actively involved in the development of the research questions, study design, trial management, and trial steering groups, writing study materials, and dissemination activities, but were not participants in the fRCT.

### Feasibility RCT methods

#### Recruitment

Participants in the fRCT were recruited through several avenues: physiotherapy caseloads and waiting lists (women’s health and musculoskeletal); newsletters and posters in healthcare and university organisations, nurseries, children’s centres, and primary schools; generic and targeted, paid social media (Facebook and Twitter) of local and national organisations.

#### Trial assessments

A web-based app was used for participant data collection during the trial, designed within the REDCap cloud (RCC) environment. Patient-reported outcome measures (PROMS) were used to rate pain intensity and medication use. These were collected fortnightly with several secondary outcome measures collected at 12 and 24 weeks to evaluate aspects such as function, health-related quality of life, and health and social care resource use [19]. Text or email reminders were sent to participants to prompt questionnaire completion. Reminders were sent once per day until the questionnaire was opened. Orthotic wear time was derived from temperature recorded using a Orthotimer® inserted into the seam of the DEFO which collected temperature data every 15 min.

#### The interventions

National Health Service (NHS) physiotherapists (*n* = 7) provided the interventions via two protocolised, remote sessions. A 60-min initial session and a follow-up session occurring 10–14 days after. Interventions comprised standard care (standardised advice and exercise) and for those allocated to the intervention group, a customised DEFO.

#### Standard care group (standardised information and exercise)

Comprised of two components:

1. Standardised advice: therapists facilitated a discussion focussed around the publicly available booklet from the Pelvic, Obstetric, and Gynaecological Physiotherapy special interest group (POGP) ‘Guidance for mothers-to-be and New Mothers: Pregnancy-related Pelvic Girdle pain’ [22].
2. Exercise programme targeted to the individual participant, chosen from a protocolised set of lumbo/pelvic exercises [19].

#### Intervention group

**Fig. 1:**
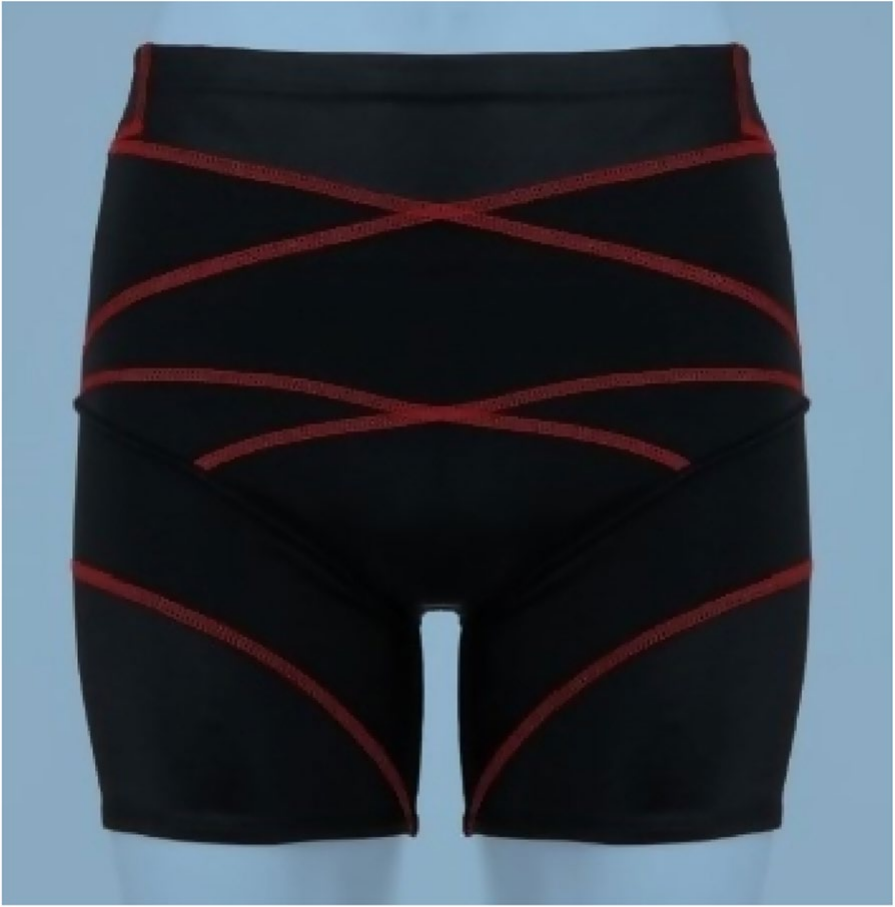
Dynamic elastomeric fabric orthoses (DEFO)

Participants received the standard care as above, in addition to the provision of two DEFO’s (Fig. 1) to be worn for 24 weeks.

### Embedded qualitative study methods

#### Research approach and methodology

Semi-structured interviews were undertaken via video conferencing, using a separate topic guide for women and the physiotherapists (Additional file 1). The topic guide centred around the aims of this qualitative study by exploring the acceptability of the trial methods, acceptability of the DEFO, impact of the intervention, and adherence to the exercise regime.

#### Sample size

The aim was to recruit ten trial participants (*n* = 5 intervention group, *n* = 5 control group) and five physiotherapists who had delivered the interventions, from across the trial sites. This has been found to be sufficient in similar studies to allow for a diversity of experience across the trial sites and arms of the study, using a purposive sampling approach [23, 24].

#### Eligibility and consent

All participants involved in the fRCT, and all physiotherapists delivering the trial interventions, were eligible to participate in the embedded qualitative study.

Written consent for involvement was sought concurrently with consent for fRCT participation. This was reviewed at the end of the fRCT (24 weeks) to ensure consent remained. Women who had not initially expressed an interest in participating in the qualitative study were invited again at 24 weeks to maximise the sampling pool available.

#### Qualitative study sampling

Purposive sampling was used to select participants, aiming to achieve maximum variation. For the fRCT participants, a sampling matrix informed the selection in terms of study site, age, parity, and duration of PPGP. All participants eligible for the fRCT, were eligible to participate in the qualitative study.

For the physiotherapists, the only variable in the sampling matrix was the study site, due to the small number of physiotherapists involved in providing the trial intervention.

#### Data collection and storage

Data were collected using teleconferencing software (Microsoft Teams/Zoom) dependent on participant preference. All interviews were recorded and stored securely in line with the study data management plan. Interviews were conducted by S.C and B.H, transcribed verbatim using an automatic transcription provided by the teleconferencing software (cross checked by two researchers B.H and S.C), dated, and anonymised. Only the participants and the researcher were present during the interviews. No field notes were taken.

#### Data analysis

Data were analysed using thematic analysis [25] according to the Braun and Clarke six-phase approach [26] in NVivo 14 (QSR International, Southport, UK). Braun and Clarke provide a clear, flexible and systematic method for identifying, analysing and reporting qualitative data. The six phase framework is widely recognised as allowing rigorous interpretation of the data and includes stages of familiarisation (transcribing), coding, theme generation, reviewing themes, naming themes and writing up [25, 26]. A pragmatic hybrid approach was taken using inductive and deductive methods in line with feasibility study objectives [27]. Trustworthiness and credibility were optimised through several strategies. Transcripts were independently coded by two members of the research team (B.H and S.C) with backgrounds in musculoskeletal physiotherapy and occupational therapy in the NHS and experience in qualitative data analysis. A summary of the main themes was sent via email to participants after data analysis for member checking to ensure credibility of findings. One participant in the control group responded confirming agreement. S.C and B.H did not have any prior relationships with the participants interviewed (were not involved in their trial interventions). To minimise bias and enhance reflexivity, detailed weekly discussions (B.H and S.C) were held on coding decisions during analysis alongside a data workshop with (J.S). In addition, discussions were held with the trial management group (TMG – see notes at end of article)—discussing recruitment progress, sample diversity and interim results, which included the lead for the qualitative sub-study (J.S) who has extensive experience in qualitative analysis.

## Results

Five physiotherapists (80% female) from the three NHS trusts, and seven women with chronic PPGP participated in the interviews; all of whom had been involved in the fRCT. Consent was gained from 17/24 women at the beginning of the trial. However, the sample size of 10 was not reached due to lack of response when contacting women at the end of the trial. The women with PPGP were recruited from across the range of participating trusts (four from site 1, one from site 2, two from site 3). Demographic information of these participants is presented in Table 1. Ages of the participants ranged from 33–47. Two participants were from an Asian ethnic background. All other participants identified as white. All physiotherapists were based in Women’s health teams except one in musculoskeletal outpatients. Interviews with women experiencing PPGP lasted between 15–40 min, and between 15–30 min with clinicians. Five participants were in the intervention arm and two in the control arm of the trial. The number of births (parity) of participants ranged from 1–5. Across the participants, there was a range of self-reported comorbidities including depression, hypermobility, Ehlers Danlos Syndrome, hypertension, migraine, and diabetes.

**Table 1:** Demographic details of participants

ID	PPGP duration	Age (years)
1	13 years	46
2	3 years	34
3	1 year 4 months	37
4	3 years 10 months	40
5	3 years 5 months	38
6	1 year	33
7	8 years 6 months	47

Legend: *ID* identifier, *PPGP* Pregnancy-related pelvic girdle pain

Four overarching themes were evident from the data 1) Acceptability of trial methods 2) Intervention acceptability 3) Impact of intervention 4) Adherence to intervention. Each theme is presented with a number of sub-themes. Data is presented combining physiotherapist (coded HCP 1–5) and participant (coded P1-7) responses.

The majority of participants were seen remotely in the fRCT, although in three cases there was a deviation from protocol wherein the intervention was delivered face-to-face rather than remotely via video conference (two cases due to poor internet connectivity and one physiotherapist deviation from the protocol without reason). All interviews were undertaken using videoconferencing.

### Acceptability of trial methods

#### Recruitment challenges and solutions

Physiotherapists were aware of the difficulties in recruiting participants to the trial. They offered valuable insight into potential avenues relevant to future trial design. Physiotherapists felt health seeking behaviour may have shifted to remote, low investment methods. E.g. not wanting to make first contact on the phone or fill out simple details in a form downloadable from the trial webpage. Of note, a trial email (advertised on all study materials) was in use, via which potential participants could register their interest; however, it was sparsely utilised. An integrated web-based form may be a useful step to encourage engagement.

“…it’s just a shame that at 6 to 8 weeks we don’t screen these ladies better with their GP appointment and then be like oh by the way, you know, if you want to access this trial this is here or actually self-refer yourself into physio because actually if we pick you up now we might prevent you really struggling in those early stages of motherhood rather than waiting a year down the line and still be struggling.” HCP2

“Physio comes way down the line as opposed to baby and their appointments so and... Yeah, it’s a difficult area because I think the biggest kind of brick wall you’re gonna come up against is the prioritising. Mums don’t prioritise themselves”. HCP4

“I’m sure they say it’s because, well, if you sent me a reminder, I probably would [come]. So, it’s having that reminder, probably at three months, and then at the end of that month. And almost I don’t want to say but annoy them a little bit to come. And then they do tend to come.” HCP4

One solution was to increase the ease to demonstrate their interest in trial participation. One physiotherapist felt that completing a form to send back in the post was too great of a demand and simple, low-time investment methods work well locally.

“Something that they can fill in on a website, even like a little questionnaire that then sends off that they can do it without having to take the paper to the post box.” HCP4

#### Data collection processes

Participants reported that they found the web-based app easy to navigate and flexible, highlighting that it made the submission of data easy.

“…the fact that it came through via text [reminder]. That was really good, you could keep it open and come back to it [PROMS], you can go, oh, you’ve got 5 minutes. I’ll just do a little bit more, which was quite good”. P2 (Control)

“I think you would probably not get anything back if it was sent via paper” P3 (Intervention)

However, for some participants finding the time to complete this was still problematic.

“…the thing I struggled with the most was just, it sounds so silly like just finding the five minutes to complete the two-week questionnaire.” P5 (Intervention)

“I think you know my lifestyle being a mom, I really don’t have time to be sitting down” P3 (Intervention)

The data collection via web-based app resulted in one participant being unsure as to what data to provide, feeling isolated with no one to ask.

“…you can’t really ask questions. So if you’re not sure how something relates to you or if there’s a bit of a question [PROMS], you kind of have to kind of go with your own gut instinct” P2 (Control)

Some clinical teams wanted trial-related data collection (fidelity checklists) to be web-based. This related to whether they used electronic or paper notes in their practice.

“I could just copy and paste it into their notes rather than repeat it by typing it again” HCP4

#### Reminders

Reminders via text positively affected data collection, however, the timing of these reminders was highlighted as an important consideration to enhance data collection.

“…a reminder for someone like me is just invaluable, I need that prompt sometimes. It’s very helpful to have that text message through with the link” P5 (Intervention)

“Yeah. I mean, you know, evenings are better for me, obviously, because babies in bed. If it arrives in the morning [PROMS], it just goes completely out the window.” P6 (Intervention)

Participants wanted a warning that the 12 and 24 weeks were a larger data collection time point requiring a greater time investment. For one participant, this led to some anxiety.

“I didn’t necessarily realise this was the biggie. It was just like here’s your fortnightly. And then I think I started it and was like, oh It’s taking a lot longer. I’m gonna have to come back to this one [PROMS] it would be better if I could plan ahead. I could feel myself getting a bit panicky like oh God, there’s another question, when’s it gonna end?” P2 (Control)

#### Frequency

Two participants struggled to provide an average score for pain intensity at the fortnightly data collection points and would have preferred a question relating only to the past week.

“I think fortnightly is quite a broad [timing of PROMS] I found that within that two-week period I had really, really good days and really, really bad days” P5 (Intervention)

“I think it would be easier to sort of estimate your pain levels on a weekly basis” P6 (Intervention)

It made one participant reflect on her situation more than she would normally do, making her more aware of the pain.

“You don’t ever have that fortnightly. Sit down. Reflect on how have things been, do you? So, I guess it makes you a little bit more aware of the pain” P2 (Control)

#### Technical issues

Several participants reported occasional technical issues with the survey links, where they were able to open the survey but not input data.

“There was once I had a problem with the survey. It was just a something that needed to reset. And then it was fine and contacting somebody was really, really easy” P6 (Intervention)

#### Virtual nature of the trial

The virtual nature of the trial had mixed views on its acceptability with physiotherapists expressing a lack of trust in the remote screening of PGP and women wanting at least one in-person intervention.

Two physiotherapists questioned the virtual nature of the screening process and eligibility criteria.

“I think the objective assessment criteria is probably not what we would use as our objective assessment. I would just want to do that objective myself, hands-on objective” HCP2.

“…are we including more of your chronic, biopsychosocial people rather than pelvic girdle pain. I know it’s chronic pelvic girdle pain but I was just a bit like 11 years right?, but certainly I think, yeah, the chance of it being pelvic girdle pain that long afterwards is no” HCP4.

There was a consistent finding that both participants and physiotherapists would have preferred an in-person element to the intervention, particularly the first session, to enhance the quality of the assessment and intervention delivery.

“…the initial assessment I think would be easier in person” P6 (Intervention)

“I think face-to-face because they were able to get me to do some movements [assessment] and give me feedback and I don’t think I’ve been able to obviously achieve that if it was done virtually” P3 (Intervention)

“…it is such a diverse group of symptoms that people get, I don’t believe you would ever get a good virtual assessment. You’re looking for so many different factors the quality of the movement, what muscles they are engaging as they’re doing that movement.” HCP2

#### Delivery of the intervention virtually

Some aspects of treatment were easier to deliver virtually (advice). Physiotherapists particularly reported difficulty in the virtual assessment of PPGP.

“It was easy in terms of the delivery [of] the advice booklet I just shared the screen” HCP1.

“…without having had my hands on someone and knowing exactly what I was specifically trying to target, it was really difficult.” HCP2

One physiotherapist reported that it was difficult to ensure a good fit of the DEFO virtually.

“You don’t know what’s normal, whether what she was feeling is just compression and doing it over a screen where I’m sort of saying can you fit a fist down them? And she’s trying to sort of hold the screen at an angle so I can see she’s got a crying baby in the background. I think as physios we’re just not geared up to do things over the phone” HCP2

On a few occasions delivery of interventions virtually was not undertaken due to the lack of reliable internet access for either the physiotherapist or participant.

“Our Internet cannot cope with us all being on it at once. So, we are slowly moving back into more face-to-face anyway. So yeah, (face-to-face) much easier cause you can have that to-and-fro conversation a little bit and make it a bit more specific to the person” HCP4.

Participants used a mix of technology (laptop/tablets), with a mix of responses.

“I think I did it once [intervention session] on the laptop and I think I once did it with the iPad and I put it on the floor and he’s like trying to get the angle to look at me and that sort of thing, and it’s so, it’s a tricky one isn’t it? Because I suppose it makes [his] job harder” P2 (Control)

“I don’t have access to a laptop or computer at home, so everything is done on a tablet or my phone. And I think I had absolutely no issues anytime” P5 (Intervention)

#### Treatment equipoise and randomisation

Participants reported no issues about the randomisation process. One participant reported that they were disappointed after being randomised to the control group. Clinician’s views of treatment equipoise were not discussed.

“…bit disappointing when you are randomised and in the control group. You don’t want to take part in research just so that you can benefit from it. But at the same time, there is a little bit of you that actually is hoping that maybe you might benefit from it” P2 (Control)

### Intervention acceptability

#### Advice

The advice component was generally acceptable to the physiotherapists, however with scope for improvement in this element to accommodate the diversity of PPGP presentations. Participants with PPGP reported limited value in the booklet. As most had experienced PPGP for several years postpartum, some had seen the booklet before.

“I didn’t regularly look at it [advice booklet], it was probably the sort of thing where I read it right at the very start and then went. Yeah that makes sense” P2 (Control)

“I think it’s an absolutely fine resource to give to patients [advice booklet]. We’ve got our own one [advice booklet], we’ve adapted it more towards pelvic floor than what the POGP stuff does.” HCP2

“You can never find a really good leaflet about it, because not everyone presents the same”. HCP2.

#### Exercise

Overall, the participants felt the personalised exercises were acceptable, however in some cases they were similar to exercises they had been prescribed before, providing some evidence of the validity of the standard care intervention provided in the trial.

“It was very similar to physiotherapy that I’ve been given before” P2 (Control)

“Having a personalized kind of exercises as well was also good” P4 (Intervention)

“You know, the advice was really good too, you know, maybe don’t push it so it hurts, but push it a little bit, you know more everyday or, you know, every couple of days. I’m just working up to a point. Very useful.” P5 (Intervention)

Physiotherapists felt the range of exercises was restrictive due to the diversity of presentations.

“PGP is kind of an umbrella term because those people are never just a simple recipe book of exercises. There’s always so many other factors built into PGP, whether it’s a DRAM (diastasis rectus abdominus muscle) in their tummy, lower back pain, or body deconditioning” HCP2.

#### Acceptability of the DEFO

There were mixed views on the acceptability of the DEFO with an almost equal number of positive and negative views. Negative views primarily focused on a lack of comfort related to wearing in warmer weather.

“The only thing I will say is with the very hot weather [the DEFO]. There were some days I was like, I can’t wear them today because it was just it was crazy hot” P6 (Intervention)

Several women reported that the DEFO provided support and would continue to wear them beyond the trial.

“I think I will continue to wear them, particularly if I’m having bad pain because I do find that if I’ve got bad back pain particularly that it [the DEFO], it does help.” P6 (Intervention)

“I’ll use the shorts [the DEFO] for doing longer walks, when I take the kids out because I think it’s taking the kids out which is the most painful thing I do. And it’s kind of holding their hands and it kind of makes me lop-sided”. P4 (Intervention)

Physiotherapists were not able to report on the acceptability of the DEFO as the two intervention sessions were protocolised within a two-week window at the beginning of the participant’s involvement in the trial. However, physiotherapists found it easy to deliver the intervention as the participants all had the DEFO ready to don on the first intervention session.

“…really easy to do because the lady had the shorts” HCP4.

Some participants required amendments to the DEFO and the physiotherapist’s capacity was an issue to deliver this in a timely manner for the participant in question. However, the process was acceptable to physiotherapists.

“I sent it back to someone else to deal with because I literally didn’t have an appointment for the next six weeks [re-measuring the DEFO] they sent me a list of you could try this and this” HCP 2

“I did have experience of this [DEFO amendment], and I had made contact through details set out in the trial file at the site, so that was fairly straightforward.” HCP1

“I think we only had an issue with one regarding her shorts, and where there was a quite a bit of back and forth to the manufacturers to try and get that sorted, but it got sorted in the end.” HCP5

#### Wear time adherence

Women reported a mixture of patterns of use, wearing the DEFO when in pain and for specific activities that aggravated their pain, such as walking and undertaking exercise.

“So, it’s not like all the time. And when I find out I’m OK and I’m not in pain, I don’t tend to wear it, and then I get in pain. And I ahh I should have worn my shorts”. P7 (Intervention)

### Impact of intervention

Despite the mixed views on the acceptability of the DEFO most comments were about the positive impact of the DEFO. This contrasted with the advice and exercise components, where minimal positive impact was reported.

#### Advice impact

Participants reported not remembering the advice booklet received, however, valued the verbal explanation of the causes of their PPGP.

“I can’t remember if I had it [the booklet], but if I had, it would have been. It would have been useful. I think I did. I think he sent me stuff.” P1 (Control)

“The [verbal] advice that they give you is so valuable and it’s so helpful and he really explained to me why I might be suffering, and in pain still” P5 (Intervention)

#### Exercise – impact

The prescribed exercises were similar to participants’ previous experience of exercises to manage their PPGP, which had not significantly helped their PPGP.

*“*Wish I could say yes, but if I’m honest and because the physiotherapy exercises were very similar to physiotherapy exercises I’ve been given previously, I don’t think I really noticed any difference” P2 (Control)

#### DEFO – impact

Participants generally reported a positive impact of the DEFO. However, some participants reported no impact. Three key subthemes were apparent (Holding it together, Confidence, and Sensory feedback). Women strongly felt that the DEFO was supportive and helped “hold them together”, preventing further damage and providing confidence to be more active. Participants also felt they provided useful sensory feedback. Some of the impact may be related to participants’ views on the biomechanical cause of their PPGP, namely instability. No participants reported a negative impact of the DEFO.

#### Holding it together

“I felt it more as like a crutch. So, if you’re in pain and you’re wearing the shorts it helps hold things together. It won’t take the pain away. It’s more to kind of like, hold things together” P7 (Intervention)

“…my physiotherapist commented that my pelvis had sort of righted itself cause my pelvis is quite twisted before the study, so again I think maybe the shorts help just to keep it in position, which was good”. P6 (Intervention)

“I do feel like they’ve kind of held me almost together, just that extra, you know, bit of support for really to kind of stop me from damaging myself further” P4 (Intervention)

#### Confidence

The DEFO provided confidence for some women which enabled a greater level of physical activity.

“…it gives you a bit more confidence some days” P6 (Intervention)

“…give a bit of confidence of walking that you know, I can just take things a little bit further than I could before. So that’s, that’s good.” P4 (Intervention)

#### Sensory feedback

Participants reported that the DEFO provided a sense of feedback. They highlighted that, because of this, the DEFO was used when undertaking the exercise programme.

“…feedback when you’re doing the exercises, I think that that’s quite helpful as well.” P4 (Intervention)

“I’m more aware that I can’t just twist anyhow, I can’t just stand up immediately, I have to take my time and I think it’s more from an awareness point of view” P3 (Intervention)

### Adherence to exercise

The level of adherence to exercise during the study varied with some participants performing exercises regularly and others forgetting to undertake them regularly, only remembering to do so when their pain levels were higher.

“I was much better at doing it when I knew I had appointments, but after the last appointment, it kind of like dwindled down, and then when I’m in pain I’m like, ahh, I should have done that, and then I’ll start doing it again and then it will start dwindling down” P7 (Intervention)

“I’m not wonderfully good at being compliant with the exercises. I mean, I do the basic pelvic floors every day but then the others are a bit more hit and miss for the exercises.” P4 (Intervention)

#### Barriers

Barriers to exercise included time constraints, and not prioritising their health over other commitments.

“So, it is hard to remember and obviously you want to prioritize your health absolutely, but it’s not that you don’t want to [exercise], life, it gets in the way” P7 (Intervention)

“After a while I wasn’t doing it as much as I should be just due to just demands... with the older kid and my baby and stuff like that” P4 (Intervention)

#### Facilitators

Facilitators to exercise included the advice provided at the intervention session, having a supportive family member, and involving their children.

“My husband was very helpful as well with times that couldn’t do any stretches because of the pain, he would encourage me. So, it felt like a lot of family was pretty much involved as well.” P3 (Intervention)

“They [the children] just do them with me” P5 (Intervention)

## Discussion

This embedded qualitative study is the first to explore the experience of participating in a remotely delivered fRCT comparing standard care with/without wearing a DEFO for managing PPGP, from the perspectives of physiotherapists and women with chronic PPGP. The findings provide important insights regarding the design of future remote trials, and the subjective experience of wearing a pelvic DEFO in the management of chronic PPGP.

### Recruitment

Recruitment to the qualitative sub-study and fRCT was a significant challenge. The physiotherapists in this study recognised that one barrier to this was that women may not prioritise their own health due to competing demands, consistent with the literature [28, 29]. They highlighted the potential importance of primary care in the recruitment process. However, several challenges exist to the effective involvement of primary care; pelvic girdle pain (PGP) does not have an ICD-11 code; the condition lacks societal acceptance and recognition by HCP’s [30] and time barriers for general practitioners [31, 32]. Targeted personal communication, such as a telephone reminder about the study following provision of the participant information sheet was one suggestion. Further research is required to understand how best to recruit women with PPGP to trials, which is currently being undertaken through the OPRiPPP (*Optimising participant recruitment and retention in studies investigating pregnancy-related pelvic girdle pain*) project led by (J.F) [33].

### Screening

A key criterion for eligibility for the fRCT was a > three-month history of postpartum PPGP with no cap on chronicity. Physiotherapists felt this wide-ranging spectrum of chronicity meant there was considerable complexity in clinical presentations. For instance, one physiotherapist could understand the presence of PPGP associated with breastfeeding but not thereafter, despite evidence of its persistence > ten years postpartum [3] and no evidence of a link between PPGP and breastfeeding [34]. Several other myths are also associated with PPGP [35]. These include beliefs that it will resolve after birth [28] and that it is a purely biomechanical condition [36] associated with joint instability, despite a growing recognition that psychosocial and physiological factors play a role in PPGP [37]. This could lead to potentially unhelpful narratives e.g. PPGP is caused by unstable pelvic joints [35], despite weak evidence [37–41].

Participants were screened remotely using self-performed pain provocation tests which have demonstrated equivalence to clinician performed tests for chronic PGP [42]. Despite this, some physiotherapists’ portrayed a lack of trust in the screening process. Beliefs held by physiotherapists’ may impact the treatment delivered [43, 44]. In this feasibility study, the treating physiotherapists’ engaged in a single, one-hour training session prior to commencement of the trial. More in-depth training of trial physiotherapists, including the justification and rationale for trial methodology choices such as screening, may enhance their trust in the various trial procedures.

### Trial delivery

Despite the trial being designed to be delivered remotely (on advice from the PPI group), both participants and physiotherapists highlighted some challenges with this delivery method and expressed a strong desire for inclusion of a face-to-face element to the intervention, consistent with other research [45]. Physiotherapists felt they were unable to provide an equivalent level of care despite, evidence that telerehabilitation appears to be equally effective in spinal care [46]. A hybrid or preference-based design [47, 48] for future studies in this population may therefore be of benefit, to ensure that there is the option of face-to-face sessions. The discussions indicated that if face-to-face delivery is offered then at least the first session should be face-to-face.

The web-based app for data collection was positively received by participants, who were thankful of the flexibility it offered, and the prompting that it provided through the system generated reminders. Despite this overall positive experience, several areas for future improvement in the reminder function are worthy of comment (Table 2). These are not only relevant for the design of a definitive trial in this population, but also for other remote trials.

**Table 2:** Challenges and solutions for remote data collection

Issues highlighted by participants	Challenges	Solutions
Consistency of reminders	Current RCC system is limited to only send reminders when a survey link has not been opened. Opening of web link but no data entry turns off the reminder function with no further reminder messages being sent. This has the potential for data loss	Explore technical solutions to improve the consistency of reminders
Timing of reminders	Pre-set option via RCC and is midnight. This was not known by the trial team. This is likely not suitable for many participants. It is not currently possible to individualise the reminder time	Set the time for reminders during trial set up phase Functionality to include individual preferences for reminder timing should be investigated. Individuality in reminder timing is possible using Ecological momentary assessment (EMA) practices [49] PPI into the design process would be advisable if a bespoke option not possible
	Individuals may have different preferred timings for reminders to be sent to maximise data collection	
Content of reminders	Text reminder message content did not highlight to participants the expected length of the survey	Adjust text message reminder text to indicate expected time to complete survey

Legend: *EMA* Ecological momentary assessment, *RCC* REDCap Cloud

Some participants reported difficulty in estimating their pain intensity on a fortnightly basis. Capturing pain intensity (worst and average, day, and night-time pain) on a fortnightly basis may increase the awareness of pain through vigilance, with a potential for increased pain intensity associated with this [50]. Tracking the trajectory of pain over a trial is however important, to identify when benefits may start to occur. Increasing the frequency of questions may, however, also have the potential to increase pain as seen in Ecological momentary assessment (EMA) methodologies [51]. The experience of participants in this study underlines the fine balance that needs to be struck so as not to enhance the focus on this. The involvement of a diverse PPI group in future would assist in developing acceptable follow-up schedules in a future trial.

### DEFO adherence

Participants described how they targeted their use of the DEFO to meet their individual needs (e.g. to enable longer walks, exercise) rather than wearing it according to the guidance provided, of 42 h per week. For many participants the DEFO was not acceptable to wear in hot conditions. A range of factors can affect the adherence to using orthotics [52], including environmental. The wear time threshold of 42 h per week used in this trial was based on DM orthotic recommendations, which is based on their clinical experience. To date, no evidence-based guidance exists to inform decisions around wear time adherence for DEFOs designed to improve PGP. Previous research of non-rigid pelvic orthotics has shown positive benefits with short periods of use [53]. Therefore, what is considered acceptable wear time adherence needs further consideration and research.

### DEFO impact

Consistently, participants felt the DEFO “held them together” which may have increased their confidence to exercise safely [53, 54] reducing the threat of damage and fear of movement, through the provision of external support and increased awareness of the body [55]. Interventions are designed to target specific mechanisms considered relevant in the condition, with DEFO’s designed to improve stability [56]. The sense that it held them together implies that the participant’s felt unstable. Whether they felt this was joint instability or related to the stability provided by muscular system was not explored in this study. Recent evidence suggests that women have beliefs that the cause of their PGP is related to ligament laxity, which may resemble a feeling of joint instability [57]. This belief may be reinforced by health care providers use of language during their consultations [36, 58–60]. Notably, however, this is not in line with current knowledge of the pathogenesis of PGP [61] and contemporary pain science [62]. Further exploration of this is important. The mechanism of action of pelvic orthotics in the management of PPGP is unknown, potentially exerting an effect through several avenues, including psychological, physiological, biomechanical and cognitive [17, 63]. Further research is needed to explore the mechanism of effect.

### Exercise adherence

The adherence to the protocolised exercises appeared low, although there should be caution in the interpretation of these results due to the recall period and risk of social desirability bias [64]. However, facilitators to exercise (e.g. supportive partner, involving a child in the exercise) were highlighted and are consistent with other research findings [65]. A key barrier to exercise adherence appeared to be a lack of contact with a physiotherapist over the 24 weeks. Whilst women’s health physiotherapists were involved in the intervention design and considered it to be usual practice in the United Kingdom, the number of sessions is not consistent with rehabilitation programme design in chronic LBP [66]. Additional contact with women over the trial is likely to be required in a future design. Further research to establish what comprises usual care for women with PPGP is important to provide greater context to the design of rehabilitation programmes. A national survey of practice and PPI would provide valuable information to assist the selection of a future trial comparator.

The diversity of the exercise programme was highlighted as limiting, despite being designed in collaboration with women’s health specialist physiotherapists. The interviews indicated that a need for a higher functional level of exercise was required. Widening the descriptor of exercise intervention to allow physiotherapists greater freedom to undertake what they consider standard care may be one way forward, as used in previous trials in low back pain [67, 68] and supported by the literature that recommends flexibility of exercise provision [69, 70].

### Strengths and limitations

The main intention of this qualitative study was to inform a future definitive RCT. The interviews highlighted important learning points, thus fulfilling its aim. Further, they highlighted trial delivery issues which may resonate more broadly with remotely delivered trials beyond this population. However, given its broad scope, some of the issues raised would benefit from deeper exploration within a more specific context, such as exercise adherence and beliefs about the DEFO impact.

This embedded qualitative study did not recruit participants to the pre-defined target. Overall, the recruitment to the fRCT was low, (*n* = 24/60, 40% of the target sample) [20]. Consequently, fewer participants were available for this qualitative study, reducing the diversity of sample characteristics between groups (control/intervention). The sample in this study were predominantly white, over the age of 30 and had more than one year duration of PPGP. It is unknown how participants would have experienced the trial, interventions or adhered to the exercise programme if they had different characteristics to the sample. A further limitation is a lack of balance in the number of people interviewed in each of the allocated study groups. Interviews were undertaken remotely, as guided by the patient and public group discussions, aiming to overcome specific barriers (caring responsibilities, employment, travel) however, this did not translate to achieving targeted numbers. Face-to-face interviews may have enabled richer data however, given the decentralised nature of this trial, and PPI preference for remote interviews, this approach was not undertaken.

## Conclusion

This qualitative study provided a deeper understanding of the experience of participating in a remotely delivered feasibility trial from the perspective of participants and physiotherapists. This provides useful information to inform future trials in this population. The remote delivery of the trial was generally acceptable, supporting the overall trial design and implementation. However, the inclusion of a face-to-face element to the intervention, suggested by both participants and physiotherapists, should be considered as a means of enhancing adherence to orthotic wear time and the exercise programme.

## Supplementary Information

Additional file 1.

Additional file 2.

## Data Availability

Data is available on reasonable request. The EMaPP study protocol and statistical analysis plan are available at https://www.plymouth.ac.uk/research/emapp-trial. Individual participant data that underlie the results will be made available (after de- identification) on a controlled access basis, subject to suitable data sharing agreements. Requests for data sharing should be made to the Chief Investigator (JAF) in the first instance.

## Abbreviations

DEFO: Dynamic Elastomeric Fabric Orthoses
EMA: Ecological Momentary Assessment
fRCT: Feasibility randomised controlled trial
HCP: Health Care Practitioner
ICD-11: International classification of diseases for mortality and morbidity statistics, 11th revision
LBP: Low back pain
NHS: National Health Service
NICE: National Institute for Health and Care Excellence
PPGP: Pregnancy related Pelvic Girdle Pain
POGP: Pelvic Obstetric and Gynaecological Physiotherapy
PPI: Patient and Public involvement
PROMS: Patient reported outcome measures
RCT: Randomised controlled trial
RCC: REDCap Cloud
RCOG: Royal College of Obstetricians and Gynaecologists
TMG: Trial Management Group
