# Supplementary material for: Participants’ and physiotherapists’ views on the delivery of a randomised controlled feasibility trial investigating a bespoke dynamic elastomeric fabric orthosis for women with chronic pelvic girdle pain: a qualitative exploration
Source: Pilot Feasibility Stud. 2026 Jun 5;12:111. doi: 10.1186/s40814-026-01847-1 (PMC13455307; doi:10.1186/s40814-026-01847-1)
Supplement: Supplementary file 2 [file 40814_2026_1847_MOESM2_ESM.docx]

# Standards for Reporting Qualitative Research: A Synthesis of Recommendations (SRQR)

| **No.** | **Topic** | **Item** | **Report location** |
| --- | --- | --- | --- |
| S1 | Title | Concise description of the nature and topic of the study identifying the study as qualitative or indicating the approach (e.g., ethnography, grounded theory) or data collection methods (e.g., interview, focus group) is recommended | Page 1 |
| S2 | Abstract | Summary of key elements of the study using the abstract format of the intended publication; typically includes background, purpose, methods, results, and conclusions | Page 2 |
| S3 | Problem formulation | Description and significance of the problem/phenomenon studied; review of relevant theory and empirical work; problem statement | Page 5 |
| S4 | Purpose or research question | Purpose of the study and specific objectives or questions | Pages 5-6 |
| S5 | Qualitative approach and research paradigm | Qualitative approach (e.g., ethnography, grounded theory, case study, phenomenology, narrative research) and guiding theory or principles; identifying the research paradigm (e.g., postpositivist, constructivist-interpretivist) is also recommended; rationale | Pages 8-10 |
| S6 | Researcher characteristics and reflexivity | Researchers’ characteristics that may influence the research process; personal attributes, qualifications/experience, relationship with participants, assumptions, and/or presuppositions; potential or actual influences that researchers may have had on the research process (e.g., biases, assumptions, and actions) and vice versa; how these were managed | Pages 10 |
| S7 | Context | Setting and salient contextual factors; rationale | Pages 6-9 |
| S8 | Sampling strategy | How and why research participants, documents, or events were selected; number of participants and rationale; why some participants opted out; when any further sampling was necessary (e.g., sampling saturation); rationale | Pages 8-9 |
| S9 | Ethical issues pertaining to human subjects | Documentation of approval by an appropriate ethics review board; informed consent; confidentiality and data security issues | Page 30 |
| S10 | Data collection methods | Types of data collected; details of data collection procedures including (as appropriate) start and stop dates of data collection and analysis, iterative process, triangulation of sources/methods, and modification of procedures in response to evolving study findings; rationale | Pages 6-10 |
| S11 | Data collection instruments and technologies | Description of instruments (e.g., interview guides, questionnaires) and devices (e.g., audio recorders); used and how they were developed; how the instruments changed over the course of the study | Pages 6-10 |
| S12 | Units of study | Number and relevant characteristics of participants, documents, or events included in the study; level of participation (could be reported in results) | Page 11 |
| S13 | Data processing | Methods for processing data prior to and during analysis, including transcription, data entry, data management and security, verification of data integrity, data coding, and anonymization/deidentification of excerpts | Pages 9-10 |
| S14 | Data analysis | Process by which inferences, themes, etc., were identified and developed, including the researchers involved in data analysis; usually references a specific paradigm or approach; rationale | Pages 9-10 |
| S15 | Techniques to enhance trustworthiness | Techniques to enhance trustworthiness and credibility of data analysis (e.g., member checking, audit trail, triangulation); rationale | Page 10 |
| S16 | Synthesis and interpretation | Main findings (e.g., interpretations, inferences, themes); might include variability of the data, integration with prior work or theory; discussion of the contribution to scholarship | Pages 10-31 |
| S17 | Links to empirical data | Evidence (e.g., quotes, field notes, text excerpts, photographs) to substantiate analytic findings | Pages 10-31 |
| S18 | Integration with prior work, implications, transferability, and contributions(s) to the field | Short summary of findings; explanation of how findings relate to and are consistent with or different from previous research or theory; main contributions of the study and its findings to scholarship and practice; discussion of applicability | Pages 24-31 |
| S19 | Limitations | Trustworthiness and limitations of findings | Page 30 |
| S20 | Conflicts of interest | Potential sources of influence or perceived influence on study conduct and conclusions; how these were managed | Page 33 |
| S21 | Funding | Sources of funding and other support; role of funders in data collection, interpretation, and reporting | Page 33 |

O’Brien, Bridget C.; Harris, Ilene B.; Beckman, Thomas J.; Reed, Darcy A.; Cook, David A.

Academic Medicine, 2014, 89(9):1245-1251, doi: 10.1097/ACM.0000000000000388
